# Residential Greenness Alters Serum 25(OH)D Concentrations: A Longitudinal Cohort of Chinese Older Adults

**DOI:** 10.1016/j.jamda.2020.04.026

**Published:** 2020-12

**Authors:** Anna Zhu, Yi Zeng, John S. Ji

**Affiliations:** a Environmental Research Center, Duke Kunshan University, Kunshan, Jiangsu, China; b Center for the Study of Aging and Human Development, Duke Medical School, Durham, NC, USA; c Center for Healthy Aging and Development Studies, National School of Development, Peking University, Beijing, China; d Nicholas School of the Environment, Duke University, Durham, NC, USA

**Keywords:** Residential greenness, normalized difference vegetation index, serum 25(OH)D, vitamin D deficiency, healthy longevity

## Abstract

**Objectives:**

Vitamin D deficiency is prevalent among older adults. We aimed to study whether residential greenness could alter serum 25(OH)D concentrations as a possible mechanism of residential greenness's positive health effects.

**Design:**

A longitudinal cohort study.

**Setting and Participants:**

We included older adults aged ≥65 years from the Chinese Longitudinal Healthy Longevity Survey (CLHLS) with follow-up between 2012 and 2014.

**Methods:**

We measured residential greenness by calculating annual average Normalized Difference Vegetation Index (NDVI) in a 500 m radius by using satellite images around each participant's residential address. Serum 25-hydroxyvitamin D (25(OH)D) concentration was dichotomized into 2 categories: nondeficiency (≥50 nmol/L) and deficiency (<50 nmol/L). We used the generalized estimating equation to examine the relationship between annual average NDVI and serum 25(OH)D.

**Results:**

We included 1336 participants in our analysis. The annual average NDVI was 0.49, and mean serum 25(OH)D was 43 nmol/L at baseline. Each 0.1-unit increase in annual average NDVI was associated with a 13% higher odds of vitamin D nondeficiency [95% confidence interval (CI): 1.01, 1.26]. The association was stronger among men [odds ratio (OR): 1.17, 95% CI: 1.02, 1.35] than women (OR: 1.08, 95% CI: 0.91, 1.29) and also stronger among those who were free of activities of daily living (ADL) disability at baseline (OR: 1.12, 95% CI: 1.00, 1.25). During the follow-up period, the participants who lived in greener areas were more likely to have an improved, rather than stable or deteriorated, vitamin D status (OR: 1.94, 95% CI: 1.51, 2.51).

**Conclusions and Implications:**

Our study suggests that higher levels of residential greenness are associated with higher serum 25(OH)D concentrations, which has implications for prevention of vitamin D deficiency among older adults.

Vitamin D deficiency [serum 25-hydroxyvitamin D (25(OH)D) <50 nmol/L] is a worldwide health problem, affecting more than 50% of the world population.^1^ Vitamin D is a critical component of cellular processes that regulate hundreds of genes, with relevance for many diseases and mortality.^2^ Low vitamin D status was shown to be related to higher risks of osteoporosis and muscle weakness,^3^ hypertension,^4^ diabetes,^5^ cardiovascular diseases,^6^ cancer,^7^ and mortality among older adults.^8^

Humans obtain vitamin D from ultraviolet exposure, food, and supplements.^9^ Ultraviolet B exposure from sunlight promotes the cutaneous production of vitamin D, contributing as much as 90% of vitamin D supply in the body.^9^ This process is affected by season, latitude, skin pigmentation, and sunscreen use. Summer and lower latitude are related to higher vitamin D concentrations while pigmented skin and sunscreen use are associated with lower vitamin D production.^10^ In addition, aging is also an essential determinant of vitamin D production, because older adults experience reduced ability to produce vitamin D, along with decreased calcium absorption, renal production of 1,25(OH)2D in the kidney, and also less outdoor activities for cutaneous production through ultraviolet exposure.^11^

Large-scale population-based cohorts in Asia, Europe, and North America have illustrated the protective effects of residential greenness on mortality,^12^^,^^13^ and there remains a need to test out potential biological mechanisms. A large consortium of cohort studies from Europe and the United States has shown that higher levels of serum 25(OH)D were associated with lower mortality rates.^14^ These findings indicate that vitamin D may be the mediator between residential greenness and mortality. To our knowledge, the relationship between residential greenness and serum 25(OH)D concentration is hypothesized but unproven. We aimed to determine whether residential greenness and changes in greenness could alter serum 25(OH)D concentrations. This process could be mediated by outdoor activity and subsequent cutaneous production of vitamin D. We tested the hypothesis among Chinese older adults aged ≥65 years by using the Chinese Longitudinal Healthy Longevity Survey (CLHLS). Vitamin D deficiency is prevalent among Chinese older adults. The 2010–2013 China National Nutrition and Health Survey (CNNHS) found that 34.1% of men and 44.0% of women aged ≥60 years reported vitamin D deficiency.^15^ Our study may provide evidence on the prevention strategy for vitamin D deficiency among older adults globally.

## Method

### Study Population

Established in 1998, CLHLS was designed to explore the determinants of healthy longevity among Chinese older adults. With a multistage, stratified sampling design, the survey recruited the participants from 22 of 31 provinces in China. Initially, the survey only interviewed the oldest old, aged ≥80 years in the 1998 and 2000 survey, and then expanded to the older adults aged 65-79 years since the 2002 survey. The CLHLS recruited new participants and conducted follow-up surveys in 2000, 2002, 2005, 2008, 2012, 2014, and 2018. In each survey, the CLHLS collected extensive data on demographic characteristics, socioeconomic status, lifestyle, physical health, and psychological well-being.^16^

Since 2008 wave, the CLHLS collected blood samples in the longevity areas, including Xiayi County in Henan Province, Zhongxiang City in Hubei Province, Mayang County in Hunan Province, Yongfu County in Guangxi Province, Laizhou City in Shandong Province, Chengmai County in Hainan Province, and Sanshui District (Foshan City) in Guangdong Province. Rudong County in Jiangsu Province was included in the 2011-2012 survey.^17^ These longevity areas covered diverse geographic regions in China. The medical professionals extracted 5 mm of fasting venous blood from the voluntary participants.

We used the 2012-2014 survey data and biomarker data on serum 25(OH)D concentrations. There were 2439 participants with biomarker data in the 2012 survey. We excluded the participants if they were missing serum 25(OH)D concentrations in 2012 survey (n = 130), were dead or lost to follow-up before 2014 survey (n = 888), and were aged <65 years (n = 85). Our final sample consisted of 1336 participants.

### Greenness Assessment

We calculated the Normalized Difference Vegetation Index (NDVI) to reflect residential greenness. During the process of photosynthesis, the plants absorb red visible light while the leaves reflect near-infrared light to scatter extra heat.^18^ NDVI is the ratio of the difference between the near-infrared region and red visible reflectance to the sum of these 2 measures. NDVI ranges from −1.0 to 1.0, with larger values indicating higher vegetation density.^19^^,^^20^

Based on the participants' residential addresses, we obtained NDVI values from the Moderate-Resolution Imaging Spectro-Radiometer (MODIS) in the National Aeronautics and Space Administration's Terra Satellite.^21^^,^^22^ MODIS has a temporal resolution of 16 days. We calculated 2 NDVI values for January, April, July, and October between 2012 and 2014, to reflect seasonal variations. We calculated the annual average NDVI values in the 500-m radius around the residence in 2012 and 2014, and cumulative NDVI values between 2012 and 2014.

### Serum 25(OH)D Assessment

We measured the serum 25(OH)D concentrations to indicate vitamin D status. Because serum 25(OH)D could reflect sources of vitamin D from both sun exposure and diet, it is thought to be the best biomarker of vitamin D status.^23^ After sample collection, the blood was stored in heparin anticoagulant vacuum tubes and centrifuged at 20°C, 2500 revolutions per minute for 10 minutes. The plasma was isolated and frozen at −20°C, shipped, and stored at −80°C in the lab. Serum 25(OH)D was assessed by an enzyme-linked immunosorbent assay (Immunodiagnostic Systems Limited, Bolton, UK). The inter- and intraassay coefficients of variation were less than 10% and less than 8%, respectively.^24^

We dichotomized vitamin D status to deficiency (<50 nmol/L), as the reference group, and nondeficiency (≥50 nmol/L).^25^^,^^26^ We categorized changes in vitamin D status during the follow-up period into stable, deteriorated, and improved vitamin D status. We defined stable vitamin D status as vitamin D deficiency or nondeficiency in both 2012 and 2014; deteriorated vitamin D status as vitamin D nondeficiency in 2012 and vitamin D deficiency in 2014; and improved vitamin D status as vitamin D deficiency in 2012 and vitamin D nondeficiency in 2014.

### Covariates

We measured a range of covariates in the 2012 survey, including season of blood draw, age, sex, urban or rural residence, education, financial support, social and leisure activity, smoking and drinking status, physical activity, and disability in activities of daily living (ADL).

Serum 25(OH)D concentrations varied by seasons, with peak values in summer.^9^ Season of blood draw was considered in the analysis to reduce the potential bias. Because the blood sample collection and interview were conducted at the same dates, the season of blood draw was categorized into spring (March, April, May), summer (June, July, August), autumn (September, October November), and winter (December, January, February), based on the months of interview. We calculated the age based on the interview dates and verified birth dates. We divided education into formal education (≥1-year schooling) and no formal education. We evaluated financial support by the question of “what is the main source of financial support.” We dichotomized the participants to financial independence if they were self-supported with their work and retirement wage, and financial dependence if they financially relied on their family members. Social and leisure activity index involved 7 activities, including gardening, personal outdoor activities excluding exercise, raising poultry or pets, reading, playing cards or *mah-jong*, listening to the radio or watching TV, and participating in organized social activities. The index was a continuous variable, and ranged from zero to 7 with each activity scored zero or 1.^27^ We categorized the participants into never smokers who did not smoke in the past or at the time of interview, former smokers who smoked in the past but did not smoke at the time of interview, and current smokers who smoked in the past and at the time of interview. We evaluated drinking status by using a similar evaluation. We defined physical activity by the question of “do you exercise or not.” We assessed ADL by using 6 self-reported questions: “Do you need assistance in bathing/dressing/toileting/transferring/eating/continence?”^28^ ADL had a continuous scale of zero to 6 with each question scored zero (without assistance) or one (with assistance). We dichotomized ADL scores to zero, defined as free of ADL disability, and 1 to 6, defined as with ADL disability.

### Statistical Analysis

We used the generalized estimating equation (GEE) to examine the association between annual average NDVI and vitamin D status from 2012 to 2014, using <50 nmol/L as the reference group. We conducted the subgroup analysis by sex and baseline ADL disability. We used logistic regression models to test whether cumulative NDVI was related to changes in vitamin D status between 2012 and 2014, using stable or deteriorated vitamin D status as the reference group. We stratified the analysis by sex. All the regression models were adjusted for season of blood draw, age, sex, urban or rural residence, education, financial support, social and leisure activity, smoking and drinking status, and physical activity. We conducted statistical analysis by using Stata, version 14.0 (StataCorp LP, College Station, TX).

### Ethical Approval

The CLHLS study was approved by the relevant Institutional Review Board. All participants signed written informed consent.

## Results

Table 1 shows the baseline characteristics of the CLHLS participants. Among 1336 older adults, the mean age was 83 (standard deviation: 11.7) years, and 47.2% were male. The mean annual average NDVI was 0.49 (standard deviation: 0.10), and serum 25(OH)D was 43 nmol/L at baseline. Compared with the participants with follow-up surveys, those who died or were lost to follow-up were older (mean age: 91 years old) and had lower serum 25(OH)D (mean concentration: 39 nmol/L) (Supplementary Table 1). Vitamin D status differed by gender, education, social and leisure activity, and smoking and drinking status (Supplementary Table 2).

**Table 1:** Baseline Characteristics, Annual Average NDVI, and Vitamin D Concentrations

Characteristics	Total	Annual Average NDVI, Mean ± SD	25(OH)D Concentrations, nmol/L, Mean ± SD
	1336	0.49 ± 0.10	43 ± 19.6
Season of blood draw, n (%)			
Spring	350 (26.2)	—	37.5 ± 18.2
Summer	915 (68.5)	—	43.6 ± 18.9
Fall	71 (5.3)	—	66.9 ± 17.2
Age, y, mean ± SD	83 ± 11.7	—	—
Sex, n (%)			
Male	631 (47.2)	0.49 ± 0.10	48.7 ± 21.7
Female	705 (52.8)	0.49 ± 0.10	38.4 ± 16.1
Residence, n (%)			
Urban area	57 (4.3)	0.34 ± 0.12	41.9 ± 15.1
Rural area	1279 (95.7)	0.50 ± 0.09	43.3 ± 19.8
Education, n (%)			
Formal education	544 (40.7)	0.48 ± 0.10	47.5 ± 21.2
No formal education	792 (59.3)	0.50 ± 0.09	40.3 ± 17.9
Financial support, n (%)			
Financial independent	372 (27.8)	0.50 ± 0.09	46.1 ± 20.1
Financial dependent	964 (72.2)	0.49 ± 0.10	42.2 ± 19.4
Social and leisure activity index, mean ± SD	2.20 ± 1.54	—	—
Smoking status, n (%)			
Never smoker	978 (73.2)	0.49 ± 0.10	41.3 ± 18.4
Former smoker	112 (8.4)	0.50 ± 0.11	45.2 ± 19.5
Current smoker	246 (18.4)	0.49 ± 0.10	50.1 ± 22.6
Drinking status, n (%)			
Never drinker	1039 (77.8)	0.49 ± 0.10	41.6 ± 18.0
Former drinker	77 (5.7)	0.48 ± 0.12	42.9 ± 17.3
Current drinker	220 (16.5)	0.49 ± 0.09	51.4 ± 25.2
Physical activity, n (%)			
Yes	209 (15.6)	0.45 ± 0.11	44.8 ± 17.2
No	1127 (84.4)	0.50 ± 0.09	43.0 ± 20.0

Table 2 reports the association between annual average NDVI and vitamin D nondeficiency between 2012 and 2014. In the fully adjusted model, each 0.1-unit increase in annual average NDVI was associated with a 13% higher odds of vitamin D nondeficiency [95% confidence interval (CI): 1.01, 1.26]. The consistent results were observed in Supplementary Table 3. The association was stronger among men [odds ratio (OR): 1.17, 95% CI: 1.02, 1.35] but not women (OR: 1.08, 95% CI: 0.91, 1.29); and also stronger among those who were free of ADL disability at baseline (OR: 1.12, 95% CI: 1.00, 1.25) than those with ADL disability (OR: 1.74, 95% CI: 0.91, 3.35).

**Table 2:** Annual Average NDVI and Vitamin D Nondeficiency From 2012 to 2014

	n	Crude	Fully Adjusted∗
All participants	1336	1.19 (1.08, 1.31)	1.13 (1.01, 1.26)
By sex			
Male	631	1.23 (1.09, 1.40)	1.17 (1.02, 1.35)
Female	705	1.11 (0.95, 1.31)	1.08 (0.91, 1.29)
By baseline ADL disability			
Free of ADL disability	1242	1.18 (1.07, 1.31)	1.12 (1.00, 1.25)
ADL disability	94	1.58 (1.01, 2.47)	1.74 (0.91, 3.35)

OR (95% CI) of vitamin D nondeficiency are shown.

∗ The fully adjusted models were adjusted for age, sex, urban or rural residence, education, financial support, social and leisure activity, smoking and drinking status, physical activity, and season of blood draw.

Table 3 presents the association between cumulative NDVI and changes in vitamin D status between 2012 and 2014. In the fully adjusted model, the participants who lived in greener areas were more likely to have improved vitamin D status, rather than stable or deteriorated vitamin D status (OR: 1.94, 95% CI: 1.51, 2.51). The protective effects were stronger among men (OR: 2.38, 95% CI: 1.64, 3.44) than women (OR: 1.59, 95% CI: 1.11, 2.28).

**Table 3:** Cumulative NDVI and Changes in Vitamin D Status Between 2012 and 2014

	n	Crude	Fully Adjusted∗
All participants	1336		
Stable or deteriorated vitamin D status	1131	Ref	Ref
Improved vitamin D status	205	2.04 (1.60, 2.60)	1.94 (1.51, 2.51)
By sex			
Male	631		
Stable or deteriorated vitamin D status	500	Ref	Ref
Improved vitamin D status	131	2.47 (1.73, 3.54)	2.38 (1.64, 3.44)
Female	705		
Stable or deteriorated vitamin D status	631	Ref	Ref
Improved vitamin D status	74	1.66 (1.18, 2.34)	1.59 (1.11, 2.28)

OR (95% CI) of improved vitamin D status are shown.

∗ The fully adjusted models were adjusted for age, sex, urban or rural residence, education, financial support, social and leisure activity, smoking and drinking status, physical activity, and season of blood draw.

## Discussion

Our study shows that higher levels of residential greenness are associated with lower odds of vitamin D deficiency among older adults. To our knowledge, this is the first study with longitudinal data to track the changes in serum 25(OH)D concentrations in relationship with residential greenness, and it provides evidence on the possible etiology in which greenness benefits health. Our analysis found that each 0.1-unit increase in annual average NDVI was associated with a 13% higher odds of vitamin D nondeficiency. We also found participants who lived in greener areas were more likely to have improved vitamin D status than stable or deteriorated vitamin D status over time. These findings indicate that maintaining or increasing greenness levels in residential neighborhoods may lead to higher serum 25(OH)D concentrations, ultimately contributing to better health for the residents.

More green space may encourage more outdoor activities^29^^,^^30^ and promote exposure to sunlight for cutaneous production of vitamin D.^9^ Studies in healthy populations found that participants who resided in areas with a higher level of greenness in Canada were more likely to participate in leisure-time physical activity.^21^ In particular, a community-level study found that living in greener environments was associated with increased walking activities, reduced usage of motor vehicles, and more active participation in recreational physical activity during the summer.^31^ In another study of rehabilitation patients, a similar consistent relationship was found—that those living in areas of higher residential greenness had greater odds of being physically active and increased post-surgical physical activity than those who lived in less green areas.^32^ If greenness does promote physical activity in our cohort, then exercising outdoors leads to more sun exposure and increases cutaneous biosynthesis of vitamin D. This can have downstream effects with interactions of serum levels of calcium, assisting in the regulation of bone metabolism; musculoskeletal, cardiovascular, and immune systems; and energy homeostasis.^33^ Additionally, we found that the effects of green space on vitamin D were only significant among those who were free of ADL disability at baseline, further suggesting that those who are able to perform physical functions and exercise are part of this mechanism.^34^^,^^35^ Furthermore, it is also possible that greenness can be associated with the availability of vitamin D in locally sourced food, as only a small percentage of the circulating vitamin D is of exogenous origin deriving from food.^36^ However, there is little evidence for this potential mechanism. Higher levels of vitamin D may explain the projective effects of greenness on cognitive function, ADL, frailty, and ultimately mortality in this cohort.^13^^,^^37^, ^38^, ^39^

Interestingly, the association between residential greenness and vitamin D status was only significant among men but not women. Women in China were reported to engage in more protective behavioral measures, such as the use of sunscreen, protective clothes, and sunglasses.^3^^,^^40^ These behaviors against sun exposure probably do not contribute to cutaneous production of vitamin D even if outdoor activities are increased in greener areas. It is also possible that men and women may have different perceptions and usage of green space, leading to different health effects of residential greenness.^41^ Our study lacked relevant information regarding the usage pattern of green space by gender. Hence, it was hard to speculate how the usage pattern would affect the association.

Our study had several limitations. First, the blood samples were collected in different seasons, while serum 25(OH)D concentrations vary according to the seasons, with peak values observed in summer. But we adjusted for season of blood draw in our analysis to reduce potential bias. Second, there may be residual confounding. Our definitions of personal characteristics, like education, were relatively rude, compared with other studies in the developed contexts. However, they reflected the special contexts in China of our participants' generation. With the mean age of 83 years at the time of the interview, our participants were mainly born between the 1900s and 1940s, when China experienced unstable and disrupted economic activities. Taking into consideration historical contexts, our personal characteristics were relatively good proxies. Our study did not have information on vitamin D supplement intake and the consumption of oily fish, which influenced serum 25(OH)D concentrations.^42^ Our definition of physical activity was not informative enough to know the time spent outdoors. It was hard to conduct mediation analysis for elucidating the mechanisms through physical activity, but we included the covariates of social and leisure activity to add supplementary information. Third, we had a portion of participants who were lost to follow-up, because of competing risks or nonresponses, or death, resulting in informative censoring. We compared the characteristics between those with and without follow-up. We found that those without follow-up were older and had lower serum 25(OH)D. Lastly, because of the advanced age of our study population, their exposure to residential greenness might be different from that of the general population. Therefore, whether the results are generalizable to younger populations remains to be seen.

The strength of our study is that it is the first of its kind to examine the effect of residential greenness on serum 25(OH)D concentrations among older adults using a longitudinal cohort. Many prior studies have shown the positive associations of residential greenness and health outcomes, and our study is able to elucidate a possible mechanism through vitamin D. Additionally, we also have a relatively large sample size of 1336 participants recruited from 8 longevity areas in China, covering a variety of climatic regions, and inclusive of both urban and rural populations. Furthermore, our findings are unlikely to be biased by the confounding effects of socioeconomic status because we found that higher levels of greenness were associated with lower socioeconomic factors in our study. This is unique from other studies in the developed countries.

## Conclusions and Implications

Our study suggests that higher levels of residential greenness are associated with higher serum 25(OH)D concentrations among older adults. The association was stronger among men than women; and stronger among those who were free of ADL disability at baseline. Vitamin D may be a mechanistic pathway in which residential greenness confers health benefits. Our findings have significant implications for prevention of vitamin D deficiency among older adults.

## Figures and Tables

**Supplementary Table 1:** Baseline Characteristics of Participants With Follow-up, and Without Follow-up Survey

Characteristics	Participants With Follow-up	Participants Without Follow-up
n	1336	888
Annual average NDVI in 2012 mean ± SD	0.49 ± 0.10	0.48 ± 0.11
Serum 25(OH)D in 2012, nmol/L, mean ± SD	43 ± 19.6	39 ± 18.7
Season of blood draw		
Spring	350 (26.2)	218 (24.6)
Summer	915 (68.5)	619 (69.7)
Fall	71 (5.3)	51 (5.7)
Age, y, mean ± SD	83 ± 11.7	91 ± 11.6
Sex		
Male	631 (47.2)	365 (41.1)
Female	705 (52.8)	523 (58.9)
Residence		
Urban area	57 (4.3)	53 (6.0)
Rural area	1279 (95.7)	835 (94.0)
Education		
Formal education	544 (40.7)	263 (29.6)
No formal education	792 (59.3)	625 (70.4)
Financial support		
Financial independent	372 (27.8)	124 (14.0)
Financial dependent	964 (72.2)	764 (86.0)
Social and leisure activity index, mean ± SD	2.20 ± 1.54	1.51 ± 1.62
Smoking status		
Never smoker	978 (73.2)	705 (79.4)
Former smoker	112 (8.4)	66 (7.4)
Current smoker	246 (18.4)	117 (13.2)
Drinking status		
Never drinker	1039 (77.8)	730 (82.2)
Former drinker	77 (5.7)	52 (5.9)
Current drinker	220 (16.5)	106 (11.9)
Physical activity		
Yes	209 (15.6)	115 (12.9)
No	1127 (84.4)	773 (87.1)

NDVI, Normalized Difference Vegetation Index.

Unless otherwise noted, values are n (%).

**Supplementary Table 2:** Baseline Characteristics of Participants by Vitamin D Status

Characteristics	Total	Deficiency, <50 nmol/L	Nondeficiency, ≥50 nmol/L	*P* Value
n (%)	1336	906 (67.8)	430 (32.2)	
Baseline annual average NDVI	0.49 ± 0.10	0.49 ± 0.09	0.49 ± 0.11	.602
Season of blood draw				<.001
Spring	350 (26.2)	270 (29.8)	80 (18.6)	
Summer	915 (68.5)	626 (69.1)	289 (67.2)	
Fall	71 (5.3)	10 (1.1)	61 (14.2)	
Age, y	83 ± 11.7	85 ± 11.8	81 ± 11.1	<.001
Sex				<.001
Male	631 (47.2)	365 (40.3)	266 (61.9)	
Female	705 (52.8)	541 (59.7)	164 (38.1)	
Residence				.850
Urban area	57 (4.3)	38 (4.2)	19 (4.4)	
Rural area	1279 (95.7)	868 (95.8)	411 (95.6)	
Education				<.001
Formal education	544 (40.7)	326 (36.0)	218 (50.7)	
No formal education	792 (59.3)	580 (64.0)	212 (49.3)	
Financial support				.062
Financial independence	372 (27.8)	238 (26.3)	134 (31.2)	
Financial dependence	964 (72.2)	668 (73.7)	296 (68.8)	
Social and leisure activity index	2.20 ± 1.54	2.08 ± 1.51	2.44 ± 1.57	<.001
Smoking status				<.001
Never smoker	978 (73.2)	695 (76.7)	283 (65.8)	
Former smoker	112 (8.4)	71 (7.8)	41 (9.5)	
Current smoker	246 (18.4)	140 (15.5)	106 (24.7)	
Drinking status				<.001
Never drinker	1039 (77.8)	733 (80.9)	306 (71.2)	
Former drinker	77 (5.7)	50 (5.5)	27 (6.3)	
Current drinker	220 (16.5)	123 (13.6)	97 (22.6)	
Physical activity				.117
Yes	209 (15.6)	132 (14.6)	77 (17.9)	
No	1127 (84.4)	774 (85.4)	353 (82.1)	

NDVI, Normalized Difference Vegetation Index.

**Supplementary Table 3:** Annual Average NDVI and Vitamin D Nondeficiency From 2012 to 2014

	OR (95% CI)	*P* Value
Model 1	1.14 (1.03, 1.26)	.013
Model 2	1.14 (1.03, 1.26)	.013
Model 3	1.13 (1.01, 1.26)	.036

CI, confidence interval; OR, odds ratio; NDVI, Normalized Difference Vegetation Index.

OR (95% CI) of vitamin D nondeficiency was shown.

Model 1 was adjusted for age, sex, urban or rural residence, education, and financial support. Model 2 was additionally adjusted for smoking and drinking status. Model 3 was additionally adjusted for social and leisure activity, physical activity, and season of blood draw.
